# Nanopore sequencing of cerebrospinal fluid of three patients with cryptococcal meningitis

**DOI:** 10.1186/s40001-021-00625-4

**Published:** 2022-01-03

**Authors:** Ke Jin, Xiaojuan Wang, Lingzhi Qin, Yazhen Jia, Keke Zhou, Yusu Jiang, Milan Zhang, Tao Zhang, Mengge Zhang, Weifeng Ma, Lin Jia, Yongshi Teng, Shuhua Dai, Wei li

**Affiliations:** Department of Neurology, Zhengzhou University People’s Hospital, 7 Weiwu road, Zhengzhou, Henan Province China

**Keywords:** Nanopore sequencing, Next-generation sequencing, Cerebrospinal fluid, Cryptococcal meningitis

## Abstract

**Background:**

Cryptococcal meningitis (CM) has a high morbidity and mortality due to the low detection of Cryptococcus in cerebrospinal fluid (CSF) during the early stage of the disease with traditional methods.

**Case presentation:**

In addition to the traditional methods of India ink staining and cryptococcal antigen (CrAg), we used nanopore sequencing and next-generation sequencing (NGS) to detect pathogenic DNA in CSF samples of three patients with CM. The CSF samples of all three patients were positive by India ink staining and CrAg. NGS also detected Cryptococcus in all three CSF samples. Nanopore sequencing detected Cryptococcus in two CSF samples.

**Conclusion:**

Nanopore sequencing may be useful in assisting with the clinical diagnosis of CM. Further research is needed to determine the sensitivity and specificity of nanopore sequencing of CSF.

## Background

Cryptococcal meningitis (CM) is an opportunistic infectious disease of the central nervous system (CNS) with high morbidity and mortality [1]. CM shows nonspecific symptoms, such as headache, fever, vomiting, seizures, and focal neurological deficits [2, 3]. With an increase in the number of immunocompromised patients with Human Immunodeficiency Virus (HIV), cirrhosis and malignancies, CM has become a public health hazard [1–5]. However, CM culture is either time-consuming or lacks sensitivity, while the detection of cryptococcal antigen (CrAg) can produce false negative results [3, 4]. Therefore, a fast and highly efficient method of diagnosis is needed.

Molecular biological methods can detect and identify pathogens rapidly and accurately without requiring culture. Polymerase chain reaction (PCR) is used in the clinical diagnosis of microbes but is limited by DNA fragment quantity. Therefore, metagenomic sequencing was used. The pathogen-positive rate of next-generation sequencing (NGS) was 28.34% in the CSF of patients with CNS infections [5–8]. In addition, Cryptococcus has a large genome. As a result, NGS has poor specificity in the detection of Cryptococcus, since it can only produce short reads with satisfactory sensitivity. In contrast, nanopore sequencing has the ability to generate long reads and real-time sequencing. Therefore, nanopore sequencing has better specificity than NGS [10, 11, 13–17]. However, the clinical application of nanopore sequencing is currently limited and further study is needed.

## Case presentation

### Patients

This study was conducted in the People’s Hospital of Zhengzhou University. The CSF samples were collected from three patients who provided signed informed consent and stored at − 80 °C from three patients who provided signed informed consent. The samples were only used for this study.

### DNA sequencing

#### NGS

DNA was extracted from the samples with the TIANamp micro DNA kit (DP316, TIANGEN BIOTECH) and sonicated to a size of 150 bp. The DNA libraries were constructed through end repair, adapter ligation, PCR amplification, and sequencing on the BGISEQ-50/MGISEQ-2000 platform. High-quality sequencing data were generated by removing short (< 35 bp), low-quality and human sequence data. The remaining data were aligned to four microbial genome databases.

#### Nanopore sequencing

DNA was extracted by a QIAamp DNA mini kit (QIAGEN 51,304, Valencia, CA, USA). The DNA libraries were constructed using a rapid PCR barcoding kit (SQK-RPB004) and sequenced on Flow Cell R9.4 or R9.5 of a MinION platform. The sequencing data were base-called in fast5 files and demultiplexed in fastq files. After removing the short and low-quality reads, the reads were aligned with the microorganism genome database. Species with identified reads higher than two for ONT data were reported.

### Clinical findings

**Fig. 1.**
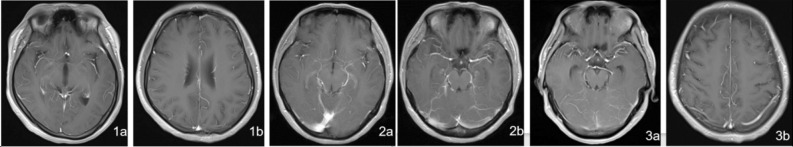
**1a** and **1b** are the axial enhanced T1WI of case I. **2a** and **2b** are the axial enhanced T1WI of case II. **3a** and **3b** are the axial enhanced T1WI of case III. The MRI showed increased meningeal enhancement in all three cases

The study included one male and two female patients, whose ages were 27, 32 and 60 years, respectively. One patient was exposed to pigeons before the onset of symptoms. All patients presented with headache, fever and stiff neck after infection. One patient experienced a seizure, while another patient had decreased level of consciousness. Medical imaging showed invasion of the meninges. CSF tests revealed elevated intracranial pressure (345–400 mmHg) and increased white blood cell (WBC) counts (1–343 × 10^6^/L). All three patients were positive by CSF India ink staining and CSF CrAg detection but negative by CSF culture (Tables 1 and 2). Magnetic resonance imaging (MRI) showed increased meningeal enhancement in all three cases (Fig. 1).

**Table 1:** Clinical presentation of the three cases

Case no.	Age	Gender	Fever	Headache	Stiff neck	Decreased consciousness	Seizure
I	27	Male	+	+	+	−	−
II	32	Female	+	+	+	−	+
III	60	Female	+	+	+	+	−

**Table 2:** Laboratory test results of the three cases

Case no.		CSF							
	India ink staining	Pressure mmHg	WBC × 106/L	CrAg detection	Protein g/L	ADA U/L	Glucose g/L	CL g/L	Culture
I	+	395	132	+	1.09	2.3	1.74	126	−
II	+	400	212	+	0.80	2.1	1.70	129	−
III	+	345	343	+	1.09	2.2	1.16	120	−

Nanopore sequencing detected Cryptococcus in two CSF samples (I and II) and NGS detected Cryptococcus in all three CSF samples. Nanopore sequencing produced 17,566 reads and 14,366 reads that were aligned to *C. neoformans*. NGS yielded 1, 17,394 and 97,979 reads that were aligned to *C. neoformans*.

### Treatment

All three patients were treated with amphotericin B, flucytosine and fluconazole for 31–46 days, and the symptoms gradually improved. Thereafter, the patients were discharged and continued 12 weeks of outpatient follow-up. The symptoms of headache and fever gradually disappeared, and the haemogram returned to normal.

## Conclusions and discussion

CM is caused by inhalation of Cryptococcus from soil, pigeon droppings and rotting vegetation [1–3]. CSF culture is the gold standard in the diagnosis of CM but is time-consuming and has a low positive rate. The India ink staining results are variable and dependent on the technician [2, 4]. The detection of CrAg always shows false negative results due to the frontal zone phenomenon [2–4]. Genetic testing has been increasingly used as a fast and highly efficient method to diagnose CM. Nanopore sequencing, a molecular biology method, is fast and can generate pathogen sequence information, such as drug resistance, to guide management [10, 11, 13–17].

In this study, three patients were clinically confirmed to have CM by positive detection using CSF culture and India ink staining. Nanopore sequencing detected Cryptococcus in two CSF samples and NGS detected Cryptococcus in all three CSF samples. Nanopore sequencing produced 17,566 reads and 14,366 reads that were aligned to *C. neoformans,* while NGS yielded 1,17,394 and 97,979 reads that were aligned to *C. neoformans*. The reads produced by nanopore sequencing were much longer than those produced by NGS. Thus, nanopore sequencing can sequence repetitive regions and structural variants, and provide comprehensive genomic information, such as the phenotype of antimicrobial resistance-encoding genes to help clinicians optimize the selection of antifungal agents for treatment (11, 12, 14, 18, 19).

This study was limited by the small sample size, and it is uncertain whether nanopore sequencing can increase the positive rate of pathogen detection. Therefore, further studies are needed.

Nanopore sequencing with improvements in accuracy could change the diagnosis of infection in the future.

## Data Availability

The data sets used and/or analysed during the current study are available from the corresponding author on reasonable request.

## Abbreviations

CM: Cryptococcal meningitis
CSF: Cerebrospinal fluid
NGS: Next-generation sequencing
CNS: Central nervous system
CrAg: Cryptococcal antigen
HIV: Human Immunodeficiency Virus
PCR: Polymerase chain reaction
WBC: White blood cell
MRI: Magnetic resonance imaging

## References

[1] Poplin V, Boulware DR, Bahr NC (2020). Methods for rapid diagnosis of meningitis etiology in adults. Biomark Med.

[2] Makadzange Azure T, McHugh G (2014). New approaches to the diagnosis and treatment of cryptococcal meningitis. Semin Neurol.

[3] Park Benjamin J, Wannemuehler Kathleen A, Marston Barbara J (2009). Estimation of the current global burden of cryptococcal meningitis among persons living with HIV/AIDS. AIDS.

[4] Góralska K, Blaszkowska J, Dzikowiec M (2018). Neuroinfections caused by fungi. Infection.

[5] Tenforde MW, Muthoga C, Callaghan A (2019). Cost-effectiveness of reflex laboratory-based cryptococcal antigen screening for the prevention and treatment of cryptococcal meningitis in Botswana. Wellcome Open Res.

[6] Tanu S, Mi M, Rajeev S (2020). Neurological worsening during treatment of an immunocompetent adult with *Cryptococcus**neoformans* meningitis. Med Mycol Case Rep.

[7] Xing XW, Zhang JT, Ma YB (2019). Apparent performance of metagenomic next-generation sequencing in the diagnosis of cryptococcal meningitis: a descriptive study.J. Med Microbiol.

[8] Beardsley J, Sorrell TC, Chen SCA (2019). Central Nervous System Cryptococcal infections in non-HIV infected patients. J Fungi (Basel).

[9] Trotter Alexander J, Alp A, Strinden Michael J (2019). Recent and emerging technologies for the rapid diagnosis of infection and antimicrobial resistance. Curr Opin Microbiol.

[10] Cozzuto L, Liu H, Pryszcz LP (2020). MasterOfPores: a workflow for the analysis of oxford nanopore direct RNA sequencing datasets. Front Genet.

[11] Kwak W, Han YH, Seol D (2020). Complete genome of *Lactobacillus**iners* KY using Flongle provides insight into the genetic background of optimal adaption to vaginal econiche. Front Microbiol.

[12] Spealman P, Burrell J, Gresham D (2020). Inverted duplicate DNA sequences increase translocation rates through sequencing nanopores resulting in reduced base calling accuracy. Nucleic Acids Res.

[13] Gargis AS, Cherney B, Conley AB (2019). Rapid detection of genetic engineering, structural variation, and antimicrobial resistance markers in bacterial biothreat pathogens by nanopore sequencing. Sci Rep.

[14] Kono N, Arakawa K (2019). Nanopore sequencing: review of potential applications in functional genomics. Dev Growth Differ.

[15] Oude MBB, Kik M, de Bruijn ND (2019). Towards high quality real-time whole genome sequencing during outbreaks using Usutu virus as example. Infect Genet Evol.

[16] Li CH, Chng KR, Boey EJH (2016). INC-Seq: accurate single molecule reads using nanopore sequencing. Gigascience.

[17] Scheunert A, Dorfner M, Lingl T (2020). Can we use it? On the utility of de novo and reference-based assembly of Nanopore data for plant plastome sequencing. PLoS ONE.

[18] Wick RR, Judd LM, Gorrie CL (2017). Completing bacterial genome assemblies with multiplex MinION sequencing. Microb Genom.

[19] Hong NTT, Nghia HDT, Thanh TT (2020). Cerebrospinal fluid MinION sequencing of 16S rRNA gene for rapid and accurate diagnosis of bacterial meningitis. J Infect.

